# Aging‐related changes in the relationship between the physical self‐concept and the physical fitness in elderly individuals

**DOI:** 10.1111/sms.13377

**Published:** 2019-04-29

**Authors:** Günter Amesberger, Thomas Finkenzeller, Erich Müller, Sabine Würth

**Affiliations:** ^1^ Department of Sport Science and Kinesiology University of Salzburg Hallein Austria

**Keywords:** development, old adults, physical activity, physical self‐concept, self‐efficacy, self‐esteem

## Abstract

The paper focuses on long‐term changes in parameters of self‐perception (ie, physical self‐concept, self‐esteem, and self‐efficacy), physical activity, and its relationship to physical fitness of healthy and active old adults. The sample of 22 physically active and healthy elderly (age *M*_*t1*_ **= 66.00) originates in an earlier skiing intervention study following a longitudinal study design with four time points of measurement over a period of 6 years. Self‐reports on physical self‐concept (PSK), general self‐esteem and self‐efficacy, and an activity index were assessed and compared to physical fitness data (VO_2max_ and muscle strength). Significant time effects (over 6 years) were obtained with respect to global physical self‐concept, endurance (PSK), and VO_2max_. Muscle strength turned out to be stable over time. The positive correlations between VO_2max_ and the corresponding self‐concept evaluation of endurance abilities diminished across the 6 years. Self‐esteem correlated with the PSK scales and VO_2max_. In contrast to our expectation, self‐esteem, self‐efficacy, and activity level hardly predicted changes in the PSK scales, VO_2max_, and physical strength. Although VO_2max_ and some parameters of the physical self‐concept declined over the 6 years, results indicate that physical self‐concept, self‐esteem, self‐efficacy, physical fitness, and physical activity display a complex pattern. The decrease in self‐perception measured by the correlation of PSK and physical fitness suggests that self‐concept of old adults is not sensitive to changes in physical fitness.

## 1 INTRODUCTION

In line with the papers of this supplement, the transition from late midlife (<65) to late life (≥65) is considered. This article addresses stability of and changes in physical self‐concept in relation to objective physical fitness, activity level, self‐esteem, and self‐efficacy.

The consistency and stability of personality traits across lifespan are constitutive issues of developmental and differential psychology.^1^ In physical activity contexts, aspects of self‐perception (ie, self‐esteem, self‐concept, self‐efficacy, or perceived physical competencies) are considered to be core issues of personality that are developed across childhood and adolescence, and, in turn, influence individuals’ physical activity behavior sustainable across their lifespan.^2^ Consequently, meta‐analyses, for example, of the relationship of physical self‐concept and physical activity focus on children and adolescents. Those data determine a medium effect size relationship between physical activity and physical self‐concept.^3^ Besides physical activity, which addresses the energy expenditure of bodily movements,^4^ physical fitness is assumed to contribute significantly to quality of life, especially when growing older.^5^, ^6^ Physical fitness can be defined as being able to carry out daily tasks through the use of cardiovascular endurance, muscular strength, or flexibility.^4^ Studies suggest that in young age, physical self‐concept and physical fitness are strongly related to each other,^7^ even stronger than physical activity.^7^ However, growing older is accompanied by a systematic decline of physical fitness.^8^, ^9^ Hence, maintaining a positive sense of physical self when getting older might be one of the most important challenges for adults engaged in physical activities.^10^ Theoretical models like the continuity theory,^11^ the model of selective optimization with compensation,^12^ and the self‐schemata approach^13^ consider aging as a process where positive self‐perception is challenged by the decline of abilities. Therefore, maintaining a sense of physical competence by using strategies like remaining in patterns of activity that were perceived constructive in the past, the use of compensation mechanisms, or the rescaling of goals is considered to contribute to successful aging. Roberts et al.,^1^ however, stated that empirical studies addressing those issues are rare with respect to participants of older ages (>60 years). In particular, the body of knowledge about changes in the physical self‐concept of old adults is scarce. An intervention study in this age‐group, however, reports positive effects.^14^ A 10 week combined strength and cardiovascular exercise program led to changes in the physical self‐concept of female subjects (*M*_*age*_ **= 66.8 years).^15^ Awick et al^16^ found effects of a 6‐month home‐based Digital Video Disc exercise program on physical self‐worth and other parameters in older subjects. Gothe et al^17^ proved the impact of exercise on basis of a hierarchical model of self‐esteem in a group of older adults (*M*_*age*_ **= 66.38 years). Changes could not be found in the general self‐esteem, but in the physical subdomains. A path model showing the relationship between physical activity, self‐efficacy for physical activity, subdomain‐level self‐perception, physical self‐esteem, and self‐esteem has been proved by Moore et al.^18^ It affirms the influence of physical self‐efficacy and physical activity on subdomains of the physical self‐worth in older adults. In one of our last studies, we addressed the change in the relationship between the physical self‐concept and physical fitness caused by an alpine skiing intervention.^19^ This study showed that elderly individuals’ global physical self and PSK sportiness are associated with VO_2max_ and concentric muscle strength. Sex differences were indicated by VO_2max_ predicting global physical self in females and physical strength (concentric muscle strength) in males. Additionally, correlations between subjective ratings and objective fitness scores increased in the skiing intervention group across intervention time, whereas this was not true for the controls.

Beneath such intervention studies addressing short‐term effects, little is known about long‐term age‐related changes in the physical self‐concept in old adults.

Different factors like life satisfaction, happiness, self‐esteem, loneliness, subjective health, and subjective age may influence the quality and stability of the self‐concept in old adults.^20^ Individuals actively try to stabilize their self, for example, by selecting information that meet their self‐perception and avoiding information that do not.^21^ According to the continuity theory,^11^ individuals benefit in terms of lifelong positive self‐perception when adhering to well‐known patterns of activities or routines, which they perceived as functional and satisfying in the past.

In the context of sport and exercise, physical activity is assumed to be the corresponding pattern of activity. Physical fitness, however, which reflects the capability to carry out daily tasks in a sufficient way, might also play a significant role in forming self‐perception at older age.

Hence, the aim of this study is to detect changes in physical self‐concepts of old adults over a span of 6 years with respect to physical activity and physical fitness. The special aspect is that the observed subjects had an active lifestyle with a high activity level. Therefore, it is of interest if healthy people with an active lifestyle are able to maintain their self‐perception and physical fitness over 6 years, and how subjective evaluations and objective fitness parameters interact.

Thus, this paper focuses the following research questions:

1. Do physical self‐concepts of elderly people change over period of 6 years?
2. Does physical fitness change in this time period and are those changes related to changes in psychological measures?
3. How stable are the PSK and physical fitness parameters over time?
4. Does the aging process of 6 years lead to changes in correlations between the physical self‐concept and corresponding parameters of physical fitness?
5. Do self‐esteem, self‐efficacy, and physical activity predict changes in physical self‐concepts or physical fitness?

## 2 Methods

### 2.1 Subjects

A total of 22 elderly people, 13 females (age *M*_*t1*_ **= 65.00, *SD*_*t1*_ = 2.70) and 9 males (age *M*_*t1*_ *=* 67.56, *SD*_*t1*_ = 4.04), were examined to estimate long‐term stability/changes in the relationship between the physical self and physical fitness (see Finkenzeller et al.). In order to ascertain age differences within the group, the sample was divided into 10 young‐olds (age *M*_*t1*_ **= 62.90, *SD*_*t1*_ **= 1.55) and 12 old‐olds (age *M*_*t1*_ **= 68.67, *SD*_*t1*_ **= 2.29). The data were collected in the context of an extensive project, which was initially designed to investigate the effect of an alpine skiing intervention in a multifaceted manner.^22^ The sample is characterized by above average values in psychological well‐being and psychosocial variables, such as life satisfaction, self‐concept, health status, and self‐efficacy.^23^ Details regarding the study design and participants are described in the introductory article within this supplement. The findings of this article are based on four measurement points, that is, t1 (pre‐test), t2 (post‐test, after twelve weeks), t3 (retention test, after 20 weeks), and t4 (follow‐up test, after 6 years). The number of cases varies between the different analyses due to missing data.

### 2.2 Measures

The physical self‐concept (PSK) scale was administered^24^, ^25^ to assess perceived global physical self‐concept (including all variables of the PSK), as well as the factor strength, endurance, and sportiness using a 4‐point Likert scale. These are the specific dimensions found for this population^19^ (see Table 1).

**Table 1:** Description of applied questionnaires

Questionnaire	Abbreviations	Dimensions	Likert scale	Cronbach's α
Physical self‐concept^30^	PSK	Global physical self‐concept	4 steps	0.87
		PSK endurance	4 steps	0.88
		PSK strength	4 steps	0.83
		PSK sportiness	4 steps	0.85
The Frankfurter self‐concept scales^26^	FSKN	Self‐esteem	6 steps	0.70‐0.86
General self‐efficacy^27^	SE	Self‐efficacy	4 steps	0.91
Activity index	AI	Physical activities like walking, biking, gardening	5 steps	n. a.

General self‐esteem was measured by the Frankfurter self‐concept scales,^26^ a 6‐point Likert scale with 6 items. General self‐efficacy was assessed by a 10‐item Likert scale.^27^

The activity index is calculated by the frequency and duration of physical activities like walking, cycling, gardening, and other activities (adopted from Stiller,^24^ Finkenzeller et al. in this supplement).

VO_2max_ was determined using a multistaged test on a cycle ergometer (ergoselect 100; Ergoline, Bitz, Germany). The protocol started at a power of 50 Watts (W) and was increased by 10 W every minute. Spirometry and ECG data were recorded using a Master Screen CPX (Viasys Healthcare, Hochberg, Germany) and the software Amedtec ECGpro 3.66 (medical Aue, Germany). The test was terminated at subjective exhaustion or occurrence of a termination criterion such as high blood pressure (for more details, see Dela et al. in this supplement). Muscle strength (M_max_ Extension) was estimated by determining the maximum torque of the right knee extensor muscles. The procedure for testing strength is reported in detail within this supplement by Pötzelsberger et al. (Table 2).

**Table 2:** Description of applied objective performance parameters

Variable	Abbreviations	Unit
Maximal oxygen consumption	VO_2max_	mL/min/kg
Maximum torque of the knee extensor muscles (right leg)	M_max_ Extension	Nm/kg

### 2.3 Statistics

All statistical analyses were performed using the software IBM SPSS Statistics for Windows (Version 23.0; IBM Corp., Armonk, NY, USA). The PSK scales are presented as means (M) and standard deviations (SD) for females and males for all four measurements pre‐test, post‐test, retention test, and follow‐up test. Pearson's product‐moment correlations were calculated between PSK scales and objective performance data. Partial correlations were used if important third variables (eg, sex) correlated with one or both of the examined variables. Due to the small sample size, 2 × 4 analyses of variances (ANOVAs) with repeated measures were calculated separately to test for changes over time (4) and the influence of sex (2) or age (2). As age and sex may be confounded in some of the models, Tables 3 and 4 provide information for a 2 × 2 cross‐classification of age and sex, to enable the reader to evaluate this more closely data. The Bonferroni tests were used for pairwise comparisons. Greenhouse‐Geisser values are reported in the case of the violation of sphericity. The level of significance was set at alpha <0.05.

**Table 3:** Descriptive of PSK scales and objective performance data of each assessment separated for age (rows) as well as for sex (columns)

Measure	Age	n_male_/n_female_	December 2008/January 2009 (t1)		April 2009 (t2)		June 2009 (t3)		April 2015 (t4)	
			Male	Female	Male	Female	Male	Female	Male	Female
PSK sportiness	Young‐olds	3/7	2.44 ± 0.43	2.82 ± 0.67	2.78 ± 0.46	2.94 ± 0.55	2.36 ± 0.34	2.96 ± 0.46	2.22 ± 0.46	2.96 ± 0.43
	Old‐olds	6/6	2.93 ± 0.13	3.06 ± 0.30	2.83 ± 0.15	3.08 ± 0.45	2.97 ± 0.28	2.97 ± 0.25	2.76 ± 0.31	2.79 ± 0.27
PSK strength	Young‐olds	3/7	3.13 ± 0.90	2.69 ± 0.45	3.07 ± 1.14	2.74 ± 0.66	2.93 ± 0.90	2.77 ± 0.48	2.93 ± 1.22	2.69 ± 0.72
	Old‐olds	6/6	3.27 ± 0.43	2.93 ± 0.69	3.17 ± 0.45	3.13 ± 0.45	3.33 ± 0.41	3.09 ± 0.52	3.00 ± 0.44	2.82 ± 0.38
PSK endurance	Young‐olds	3/7	2.08 ± 1.28	1.86 ± 0.84	2.08 ± 1.23	1.89 ± 0.72	2.08 ± 1.28	1.87 ± 1.06	1.75 ± 1.09	1.74 ± 0.93
	Old‐olds	6/6	2.50 ± 0.65	2.42 ± 0.63	2.42 ± 0.54	2.25 ± 0.91	2.46 ± 0.84	2.29 ± 0.75	1.81 ± 0.83	1.80 ± 0.69
Global physical self	Young‐olds	3/7	2.46 ± 0.52	2.53 ± 0.54	2.67 ± 0.62	2.59 ± 0.54	2.37 ± 0.60	2.64 ± 0.47	2.22 ± 0.65	2.61 ± 0.40
	Old‐olds	6/6	2.82 ± 0.19	2.78 ± 0.39	2.72 ± 0.16	2.81 ± 0.48	2.84 ± 0.32	2.80 ± 0.34	2.55 ± 0.40	2.57 ± 0.20
VO_2max_ (ml/kg/min)	Young‐olds	2/4	25.42 ± 5.16	23.40 ± 3.50	24.86 ± 5.78	24.20 ± 2.69	23.74 ± 5.91	24.49 ± 3.35	22.85 ± 2.33	20.25 ± 1.99
	Old‐olds	4/4	28.35 ± 12.17	25.22 ± 7.98	33.03 ± 6.77	30.83 ± 5.80	32.35 ± 7.52	29.44 ± 7.28	28.65 ± 8.06	27.13 ± 4.17
M_max_ extension (Nm/kg)	Young‐olds	3/7	2.07 ± 0.04	1.57 ± 0.29	2.06 ± 0.07	1.54 ± 0.23	2.16 ± 0.12	1.49 ± 0.23	1.90 ± 0.42	1.44 ± 0.28
	Old‐olds	6/6	2.32 ± 0.33	1.81 ± 0.43	2.32 ± 0.45	1.78 ± 0.47	2.39 ± 0.50	1.92 ± 0.46	2.29 ± 0.46	1.86 ± 0.62

**Table 4:** ANOVA with repeated measurements 2 (sex) × 4 (time)

Measure	Time					Sex					Time × Sex				
	*df*	*F*	*P*	η²	*1‐*β	*df*	*F*	*P*	η²	*1‐*β	*df*	*F*	*P*	η²	*1‐*β
PSK sportiness	2.05, 41.05	2.66	0.08	0.12	0.51	1, 20	1.79	0.20	0.08	0.25	2.05, 2.05	0.43	0.66	0.02	0.12
PSK strength	2.16, 43.28	1.48	0.24	0.07	0.31	1, 20	1.56	0.23	0.07	0.22	2.16, 43.28	0.48	0.64	0.02	0.13
PSK endurance	3, 60	8.21	<0.001***	0.29	0.99	1, 20	0.33	0.57	0.02	0.09	3, 60	0.79	0.51	0.04	0.21
Global physical self	3, 60	4.68	<0.01**	0.19	0.88	1, 20	0.03	0.87	0.001	0.05	3, 60	1.23	0.31	0.06	0.28
VO_2max_	1.57, 18.85	4.48	0.03*	0.27	0.63	1, 12	0.75	0.40	0.06	0.13	1.57, 18.85	0.02	0.96	0.002	0.05
M_max_ Extension	1.80, 36.06	1.28	0.29	0.06	0.32	1, 20	12.47	<0.01**	0.38	0.92	1.80, 36.06	0.28	0.73	0.01	0.51

## 3 RESULTS

1. Does the physical self‐concept of elderly people change in over a period of 6 years?

Means (M) and standard deviations (SD) of the four measurement points are depicted in Table 5. Table 3 provides M ± SD of the PSK scales and the objective performance tests separated by sex and for the group of young‐olds and old‐olds.

**Table 5:** Descriptive of PSK scales and objective performance data of each assessment

Measure	n_male_/n_female_	Dec. 2008/Jan. 2009 (t1)	April 2009 (t2)	June 2009 (t3)	April 2015 (t4)
PSK sportiness	9/13	2.86 ± 0.45	2.93 ± 0.42	2.89 ± 0.39	2.76 ± 0.41
PSK strength	9/13	2.97 ± 0.59	3.00 ± 0.62	3.03 ± 0.54	2.84 ± 0.62
PSK endurance	9/13	2.22 ± 0.80	2.16 ± 0.78	2.17 ± 0.91	1.80 ± 0.73
Global physical self‐concept	9/13	2.67 ± 0.42	2.70 ± 0.43	2.70 ± 0.42	2.53 ± 0.39
VO_2max_ (ml/kg/min)	6/8	25.62 ± 7.59	28.71 ± 6.18	28.04 ± 6.60	24.99 ± 5.79
M_max_ Extension (Nm/kg)	9/13	1.91 ± 0.43	1.89 ± 0.47	1.94 ± 0.51	1.85 ± 0.54

As shown in Tables 4 and 6, significant time effects are obtained displaying a decrease in the global physical self‐concept as well as in endurance. These significant time effects occur regardless of considering sex or age‐group as interacting factor. Pairwise comparisons (Bonferroni) illustrate that this is only caused by measurement point 4. No sex differences and no interactions of time × sex as well as time × age‐group are observed. A visual comparison of the means of male and female young‐olds and old‐olds (Table 3) does not suggest that an interaction of sex and age may confound the results of the 2 × 4 ANOVAs.

**Table 6:** ANOVA with repeated measurements 2 (age) × 4 (time)

Measure	Time					Age‐group					Time × Age‐group				
	*df*	*F*	*P*	η²	*1‐*β	*df*	*F*	*P*	η²	*1‐*β	*df*	*F*	*P*	η²	*1‐*β
PSK sportiness	1.92, 38.47	2.47	0.10	0.11	0.59	1, 20	0.79	0.39	0.04	0.14	1.92, 38.47	1.67	0.20	0.08	0.32
PSK strength	2.20, 43.93	1.32	0.28	0.06	0.28	1, 20	1.57	0.22	0.07	0.22	2.20, 43.93	0.48	0.64	0.02	0.13
PSK endurance	3, 60	7.22	<0.01**	0.27	0.98	1, 20	1.31	0.27	0.06	0.19	3, 60	1.76	0.17	0.08	0.44
Global physical self	3, 60	3.80	0.02*	0.16	0.79	1, 20	1.40	0.25	0.07	0.20	3, 60	1.72	0.17	0.08	0.43
VO_2max_	1.61, 19.27	4.83	0.03*	0.29	0.67	1, 20	4.08	0.07	0.25	0.46	1.61, 19.27	1.88	0.18	0.14	0.31
M_max_ Extension	1.82, 36.45	1.20	0.31	0.06	0.24	1, 20	5.20	0.03*	0.21	0.58	1.82, 36.45	1.07	0.35	0.05	0.22

*Note*.

*P <* * 0.05; **P <* * 0.01

- Does the physical fitness change in this time period and are those changes related to changes in psychological measures?

The objective performance data reflect the results of the PSK. Only VO_2max_ changes significantly over time and is not influenced by sex or age‐group. The decrease is mainly caused by measurement point 4. The analyses indicate no significant sex differences in VO_2max_. Age differences just fail significance (*P *=* *0.07; *η*_*p*_^2^ = 0.25). The old‐olds tend to have a higher VO_2max._

Muscle strength is proven rather stable over time. Sex [*F*(1, 20) = 12.47, *P *<* *0.01, *η*_*p*_^2^ = 0.389] and age [*F* (1, 20) = 5.20, *P *=* *0.03, *η*_*p*_^2^=0.21] differences in strength are substantial (see Tables 4 and 6). Again, the old‐olds are stronger than the young‐olds. This applies for male and female participants (see Table 3).

Changes in physical fitness from t3 to t4 do not correlate with changes in the psychological measures (−0.07 ≤ *r *≤* *0.34) significantly.

- How stable are the PSK and physical fitness parameters over time?

Regarding stability of person characteristics, the correlations between the measurement points (t1‐t2, t2‐t3, t3‐t4) of the PSK scales are significant (0.62 ≤ *r *≤* *0.93). The objective performance parameters VO_2max_ and M_max_ are also very stable over time (0.68 ≤ *r *≤* *95) (see Table 9).

- Does the aging process of 6 years lead to changes in correlations between the physical self‐concept and corresponding parameters of physical fitness?

Due to sex differences, the correlations have been calculated separately (Table 7A). In order to report the correlations for the whole sample, partial correlations controlling for sex have been used (Table 7B). The results reflect that the correlations between VO_2max_ and PSK scales tend to increase from measure point 1 to measure point 3, particularly with respect to VO_2max_ and the global physical self‐concept PSK, as well as the subdomains of endurance and sportiness. This effect diminishes at measure point 4 (see Table 7A and B). This is statistically supported as correlations only reach significance at time point 2 or 3. Fishers’ z however fails significance due to the small sample size. No significant correlations were found for the PSK strength parameters at all; hence, those correlations are not reported in Table 7.

**Table 7:** Pearson's and partial correlations of the PSK with physical fitness (VO_2max_ and M_max_ Extension)

(A) Pearson's correlations (male, female)							(B) Partial correlations (controlling sex)					Global physical self‐concept
			PSK sportiness	PSK endurance	PSK strength	Global physical self‐concept			PSK sportiness	PSK endurance	PSK strength	
VO_2max_		n	*r*	*r*	*r*	*r*	VO_2max_	*df*	*r*	*r*	*r*	
Male	t1	9	−0.01	−0.11	−0.20	−0.06	t1	18	0.12	0.08	0.08	0.08
	t2	9	0.07	0.16	0.05	0.09	t2	18	0.41	0.46*	0.34	0.46*
	t3	6	0.86*	0.45	0.33	0.80	t3	11	0.80***	0.56*	0.67**	0.77**
	t4	9	0.31	0.19	−0.06	0.20	t4	18	0.36	0.42	0.08	0.31
Female	t1	12	0.20	0.29	−0.05	0.19						
	t2	12	0.61*	0.61*	0.62*	0.70*						
	t3	8	0.64	0.80*	0.90*	0.77*						
	t4	12	0.30	0.50	0.15	0.45						

*Note*.

*P <* * 0.05; **P <* * 0.01; ***P <* * 0.001

- Do self‐esteem, self‐efficacy, and physical activity predict changes in the physical self‐concept or physical fitness?

Self‐esteem remains stable over time and does not differentiate between age‐groups or sex (an ANOVA with repeated measurements shows no significance). In addition, substantial correlations with the global physical self‐concept (*r*_*t1*_ = 0.58, *p*_*t1*_ = 0.004; *r*_*t2*_ = 0.61, *p*_*t2*_ = 0.003; *r*_*t3*_ = 0.53, *p*_*t*3_ = 0.01; *r*_*t4*_ = 0.52*, p*_*t4*_ = 0.05) and its subdomains as well as with VO_2max_ at t2 and t3 (*r*_*t2*_ = 0.46, *p*_*t2*_ = 0.04; *r*_*t3*_ = 0.74, *p*_*t*3_ = 0.003) are obtained.

Table 8 presents the correlations between self‐esteem, self‐efficacy, and activity level at time point 3 and the changes in the PSK scales and the objective performance data from t3 to t4 (t4 minus t3).

**Table 8:** Correlations between self‐esteem, self‐efficacy, and activity level (t3) with changes in the PSK scales and objective performance date (t4 minus t3), n =* *22 (PSK scales and M_max_ Extension); n = 14 (VO_2max_)

	Differences t4‐t3					
	Global PSK	PSK endurance	PSK strength	PSK sportiness	VO_2max_	M_max_ Extension
	*r*	*r*	*r*	*r*	*r*	*r*
Self‐esteem t3	−0.19	−0.05	−0.63**	−0.09	−0.38	0.15
Self‐efficacy t3	−0.27	−0.42*	−0.27	−0.25	−0.42	−0.11
Activity level t3	0.25	−0.04	0.08	0.30	−0.41	−0.02

*Note*.

*P <* * 0.05; **P <* * 0.01

Low self‐esteem (t3) predicts changes in the strength of the PSK scale for the overall group and for females. It is notable that self‐esteem also correlates negatively with VO_2max_ in all groups (−0.75 ≤ *r* ≤ −0.28). However, due to the small sample sizes these correlations do not reach significance.

Self‐efficacy is considerably stable over time displaying no significant sex and age differences. It is significantly associated with the global physical self‐concept at the measure points 2, 3, and 4 (*r*_*t1*_ = 0.39, *p*_*t1*_ = 0.08; *r*_*t2*_ = 0.53, *p*_*t2*_ = 0.01; *r*_*t3*_ = 0.70, *p*_*t*3_ = 0.000; *r*_*t4*_ = 0.69*, p*_*t4*_ = 0.003), but not to the objective performance data.

Self‐efficacy predicts negative changes in the PSK scale endurance (for the whole group), in the PSK scale strength (for the female group), and in the VO_2max_ parameters (for the female group).

The activity index remains unaffected over time. There is a small decline of activities in the old‐old age‐group, however failing to reach statistical significance. Thus, the activity index does not explain changes in the physical self‐concept and in the objective performance data.

## 4 DISCUSSION

This paper addresses stability and changes in the physical self‐concept in relation to physical fitness, self‐esteem, self‐efficacy, and activity level. This study has a strong explorative perspective; yet, due to the small sample size and complex relationships, results must be treated with caution.

1 and 2 Does the physical self‐concept and physical fitness of elderly people change over a period of 6 years?

The results show a significant decline in the global physical self‐concept and in the subscale endurance over the last 6 years (between measurement point three and four). This goes along with a reduction of VO_2max_. Age and the change in VO_2max_ are slightly correlated *r *= −0.41 (*P *=* *0.06, n =* *21). Muscle strength remains stable over the 6 years. Within‐group sex and age differences are significant (see Tables 4 and 6). As expected, women have lower values, whereas in contrast to our expectation, the old‐olds outperform the young‐olds. From the physical fitness point of view, these results reflect only partly the normal decline that goes along with aging. One might expect not only a decrease in VO_2max_, but even more a decrease in muscle strength.^28^ However, the latter might be a result of lifelong physical activity. The participants of this study all displayed a physical active lifestyle over years and can be assumed as long‐life physical active people. Activities such as alpine skiing might contribute to the stability of muscle strength, even 6 years later. By means of self‐perception, growing older obviously is reflected by an overall decline of perceived physical competencies, particularly with respect to endurance abilities. The subjective ratings of endurance are compatible to the objective decrease in VO_2max_ and thus can be interpreted as a realistic view of what happens to the endurance domain when growing old.

3 How stable are the PSK and physical fitness parameters over time?

The correlations of the parameters over the four measurement points indicate fairly high stability of personal characteristics. Although slight changes in terms of performance parameters or self‐perceptions were observed, the physical self‐concept as well as the objective performance parameter VO_2max_ and M_max_ is rather stable over the four measurement points. In particular, the correlations over the 6‐year span indicate the stability of the self‐concept and physical fitness parameter (see Table 9). The comparatively high correlations also indicate that the participants of the study performed the tests with high motivation and completed the questionnaires carefully.

**Table 9:** Pearson's correlations of the PSK and objective performance data of the four measurement points

		PSK sportiness	PSK endurance	PSK strength	Global physical self‐concept			Vo_2max_	M_max_
	n	*r*	*r*	*r*	*r*		n	*r*	*r*
t1‐t2	22	0.82**	0.89**	0.68**	0.89**	t1‐t2	21	0.68*	0.95**
t2‐t3	22	0.68**	0.88**	0.93**	0.81**	t2‐t3	14	0.94**	0.95**
t3‐t4	22	0.91**	0.88**	0.62**	0.85**	t3‐t4	14	0.86**	0.87**

*Note*.

*P <* * 0.05; **P <* * 0.01

4 Does the aging process of 6 years lead to changes in correlations between the physical self‐concept and corresponding parameters of physical fitness?

Due to the active lifestyle, it could be expected that the achieved competence in rating the own physical fitness measured by the physical self‐concept could be maintained or even increased. The results suggest that the correlations between VO_2max_ and PSK scales only increase form measure point 1‐3. In the 6‐year period, the correlation diminished. However, the values of the PSK scales at t3 and t4 both correlate to the same extent with VO_2max_ at t3. It is assumed that the physical self‐concept is not actualizing the decline in physical fitness across the years. This could be the result of a delay effect: In order to maintain a positive self‐perception, the subjective evaluation of competencies depends on former objective performance, and the curve of subjectively perceived decrease is substantially flatter than the curve of objective performance. Yet, due to the high variance within the data it can be expected that some participants have a realistic perception of their changes in physical fitness while others either over‐ or underestimate those changes. Further studies should look into the possible causes of these differences.

5 Do self‐esteem, self‐efficacy, and physical activity predict changes in the physical self‐concept or physical fitness?

Self‐esteem and self‐efficacy as higher order variables are stable over time do not differentiate between age‐groups or sex, and are highly correlated with the global physical self‐concept and its subdomains. They also correlate significantly with VO_2max_ at t2 and t3.

It could be expected that high self‐esteem, high self‐efficacy, and a high activity level at time point 3 predict positive changes or at least stability of the physical self‐concept and physical fitness. However, in contrast to our expectation the correlations between self‐esteem, self‐efficacy, and changes in the PSK scales, VO_2max_, and muscle strength are inconsistent, and if the correlations are substantial, those correlations are negative (see Table 8). Yet, due to the small sample size interpretations of those differentiated effects are vague. One argument for the unexpected negative correlations can be a statistical regression to the mean. When looking at the data in more detail, it is obvious that participants with a low level in the PSK or physical fitness parameters at t3 decline less than those with initial higher values at t3.

On the other hand, it could be possible that dissatisfaction leads to a change in effort.

The good health and high quality of life of the target group are demonstrated by the fact that the activity index does not change over the 6‐year period. At first glance, it is surprising that the activity index does not explain changes in the physical self‐concept and in the objective performance data.

This study focused on healthy people and demonstrated the high stability of physical and psychological parameters over 6 years in the sixth and seventh decade of living (see Table 9). In other words, as long as individuals of higher age are able to endorse an active lifestyle, psychological and physical resources are still available, enabling high quality of life.

## 5 PERSPECTIVE

We still know very little about which particular parameters come into play to reach these golden years, and how activity, health and fitness status, self‐concept, and other issues influence each other in this long‐lasting process.

One crucial question will be the influence of the quality of the self‐perception of physical fitness on health. Our results suggest people may over‐ or underestimate their changes in physical fitness. It would be of high interest to find out if this effects the physical activity level and/or the health status and how this is related to higher order variables like self‐esteem.

Hence, further studies with larger sample sizes should address those interacting processes in more detail. In particular, longitudinal data with interventions are indispensable in order to determine causal effects. Large surveys like the Whitehall II study^29^ show, for instance, that growing old is a complex scenario where parameters like physical activity, social context, cardiovascular diseases, psychological health status, and live style interact very dynamically over the years.

One of the main challenges, however, will be to cope with dropouts or to avoid dropouts to have a representative sample that display different paths of aging.

## CONFLICTS OF INTEREST

The authors declare no conflicts of interest.
